# Exploring Drivers of Children’s Food Choices: A Multi-Source Process Evaluation of a School-Based Nutrition Education Program

**DOI:** 10.3390/foods15111832

**Published:** 2026-05-22

**Authors:** Mariusz Jaworski

**Affiliations:** Department of Education and Research in Health Sciences, Faculty of Health Sciences, Medical University of Warsaw, 00-581 Warsaw, Poland; mariusz.jaworski@wum.edu.pl

**Keywords:** food choice, nutrition education, school-based intervention, behavior change, experiential learning, process evaluation, children, self-efficacy

## Abstract

Children’s food choices are shaped early in life through cognitive, social, and environmental influences, yet relatively little is known about how school-based nutrition education supports these processes in routine settings. This study examined mechanisms potentially relevant to children’s food choices using a multi-source process evaluation of the municipal “I Know What I Eat” program implemented in Warsaw primary schools. A prospective observational implementation study was conducted in 81 public schools, covering 198 workshop cycles for students aged 8–9 years. Data were obtained from teacher-observers (*n* = 198), trained program implementers (*n* = 6), and implementation records. The evaluation focused on implementation quality, fidelity, acceptability, and mechanisms relevant to food-related decision-making. Quantitative data were analyzed using descriptive statistics, Kruskal–Wallis tests, and Spearman correlations; qualitative comments were examined using content analysis. The program was implemented with high quality and consistency, with mean ratings ranging from 4.88 to 4.96 on a five-point scale and no significant differences by implementer or class size. Qualitative findings indicated that experiential learning, practical food preparation, peer interaction, and active participation supported children’s engagement. These findings suggest that school-based nutrition education can create conditions relevant to food-related decision-making, although direct behavioral measures are needed.

## 1. Introduction

Unhealthy dietary habits among children and adolescents constitute one of the key challenges in contemporary public health [1]. In many countries, the prevalence of overweight and obesity in the pediatric population remains high, accompanied by metabolic disturbances, reduced physical fitness, and an increased co-occurrence of other non-communicable diseases in adulthood [2]. At the same time, the diets of many children frequently deviate from current dietary guidelines, being characterized by excessive consumption of highly processed foods and simple carbohydrates, alongside insufficient intake of vegetables, fruits, and whole-grain products [3]. Additionally, dietary patterns formed in childhood can be conceptualized as the early development of food-related consumer behaviors, as children begin to function as emerging consumers within the food system. This period is also critical for the formation of taste preferences, purchasing habits, and decision-making patterns that may persist into adulthood and influence long-term dietary choices [4], as well as affect individual health outcomes and the burden on healthcare systems [1,2]. These early dietary patterns also form the basis of later food choices. They are shaped by cognitive, social, and environmental influences that affect how children select, accept, and consume foods in everyday settings. These early-formed patterns may also have long-term implications for food demand and dietary trends at the population level, linking individual food choices with broader food system dynamics. From a consumer behavior perspective, children can be conceptualized as emerging actors within the food system, whose early experiences shape future food preferences, purchasing patterns, and long-term demand trajectories.

Conceptually, these domains can be linked as nested and interacting levels of food-related decision-making. Dietary behavior represents the broadest level and refers to habitual patterns of food-related practices, including what, when, where, and how children eat in everyday contexts [4,5]. Food choice is a more specific component of dietary behavior and refers to the selection, acceptance, rejection, and consumption of particular foods within these broader behavioral patterns [5]. From a consumer behavior perspective, food choices can be understood not only as nutritional decisions, but also as decisions shaped by preferences, perceptions, social norms, learned competencies, food availability, environmental cues, and the broader food system [4,5,6,7,8]. In childhood, these processes are still developing and are strongly influenced by parents, peers, schools, and everyday food environments [4,5,6,7,8]. Therefore, children may be conceptualized as emerging consumers whose early food-related experiences contribute to the formation of preferences, decision-making patterns, and future consumption trajectories. From this perspective, school-based nutrition education may be interpreted not only as a health promotion strategy, but also as an intervention targeting early cognitive, social, behavioral, and environmental drivers of food choice and emerging consumer behavior [5,6,7,8].

Educational settings (e.g., schools) represent structured food environments in which early consumer behaviors are shaped and can substantially influence the development of children’s taste preferences, their food choices, and long-term consumption patterns, thereby shaping dietary behaviors and food-related decision-making. Schools are places where children spend a considerable amount of time, and the meals provided there should support their growth and development [9,10,11]. In this context, the school can be conceptualized as a key setting for health behavior formation, in which educational, social, and environmental factors jointly contribute to the key drivers of children’s food choice behaviors.

It should be emphasized that shaping children’s dietary behaviors in the school environment depends not only on the quality of the lunch meal options offered, but also on educational activities that strengthen students’ nutrition-related competencies. Appropriately designed nutrition education increases acceptance of healthier foods, supports informed food choices, and promotes the consolidation of healthy dietary habits both within and beyond the school setting [6,9]. Therefore, early prevention implemented in schools is considered one of the most effective approaches to health promotion [6]. It is also important to highlight that nutrition education in the school setting may operate through various behavior change mechanisms, such as modeling, reinforcement of social norms, the development of self-efficacy, and experiential learning [5,7,8]. These mechanisms can be interpreted as key drivers of food choice, influencing not only knowledge acquisition but also food preferences, acceptance, and decision-making processes. Understanding these mechanisms is crucial for designing effective interventions aimed at achieving sustained changes in dietary behaviors.

Previous research on school-based nutrition interventions has primarily focused on evaluating health outcomes, whereas less attention has been paid to implementation mechanisms and the processes through which educational interventions may influence dietary behaviors [12,13], as well as to the population-level effects of nutrition policies targeting schools [14]. The recent literature increasingly emphasizes the importance of process evaluation in the context of school-based nutrition interventions. This approach enables the assessment of implementation quality, fidelity to the intervention protocol, acceptability of the activities, and the identification of barriers and facilitators of effective implementation [15].

This approach helps explain how interventions function in practice and supports their refinement and scaling within educational systems and nutrition policies [16]. Process evaluation thus represents a key tool for identifying intervention mechanisms and understanding how educational components translate into potential behavioral change [17].

Despite the growing number of school-based nutrition education programs, there is a lack of multi-source implementation evidence on how such interventions are delivered at scale and how they activate underlying drivers of food choice in children, particularly in terms of mechanisms shaping food-related decision-making processes [18]. One such municipal nutrition education intervention is the “I Know What I Eat” program implemented in Warsaw [19], which is delivered as part of routine municipal educational practice.

The implementation model of this program is based on trained program implementers, which supports capacity building among staff and increases the potential for scaling the intervention. The program has a standardized structure that includes both theoretical and practical modules delivered according to uniform lesson plans, enabling the assessment of implementation quality and fidelity. An important distinguishing feature of the program is the close collaboration between the scientific community, practitioners, and local government units during both the design and implementation phases of the “I Know What I Eat” program [19]. This partnership has enabled the integration of current scientific knowledge with practitioners’ experience and the organizational context of the education system. The program represents an example of an educational intervention aimed not only at knowledge transfer but also at shaping competencies and dietary behavior patterns within the school environment. In this study, these mechanisms were not directly measured but were explored indirectly through process evaluation indicators, including student engagement, acceptability, and the role of experiential components.

The aim of the study was to conduct a multidimensional evaluation of the implementation and delivery of the municipal nutrition education program “I Know What I Eat,” with particular emphasis on identifying potential drivers of children’s food choices and the mechanisms through which the program may influence food-related decision-making processes.

## 2. Materials and Methods

### 2.1. Study Design

The study was designed as a prospective observational implementation study [20,21] aimed at evaluating the implementation process of the municipal nutrition education program “I Know What I Eat.” The study was informed by implementation research and examined how the intervention was delivered under routine conditions within the school system. The manuscript was prepared in accordance with the TREND (Transparent Reporting of Evaluations with Nonrandomized Designs) guidelines [22], and all recommended reporting elements were incorporated into the study description. The description of the intervention was developed in line with the TIDieR (Template for Intervention Description and Replication) checklist to ensure transparency and facilitate replication of the study [23].

In this study, a process evaluation approach was used to explore potential mechanisms and drivers underlying children’s food choice behaviors, with a particular focus on how the intervention may activate processes related to food-related decision-making, rather than to assess direct behavioral outcomes.

The study was conducted between 1 April 2025 and 31 March 2026. A process evaluation methodology was applied in accordance with the Medical Research Council guidance for the evaluation of complex public health interventions [24]. The aim of the process evaluation was to assess implementation quality, implementation fidelity, acceptability, and to identify facilitators and barriers to implementation. The analysis was based on multi-source data collected from key stakeholders involved in the delivery of the program.

The study was non-experimental and did not involve randomization or manipulation of intervention conditions. All participants received the same intervention within a single-group design under usual school conditions. To enhance consistency and reduce potential bias, standardized intervention protocols, uniform training of program implementers, and consistent implementation procedures across schools were applied. Blinding was not feasible due to the nature of the educational intervention; therefore, participants, program implementers, and outcome assessors (teacher-observers) were aware of the intervention. Participation was voluntary at the institutional level, with no randomization or comparison group included in the study design.

Because the evaluation used anonymized data generated during routine educational program monitoring and did not involve individual-level clinical data, medical procedures, or experimental manipulation, it was not classified as a medical experiment or clinical trial requiring formal bioethics committee approval under the Polish legal framework governing bioethics committee review.

Following the completion of the workshop cycle, a focus group with program implementers was conducted. During this session, qualitative data were collected on their experiences related to the course of the intervention, its reception by students, and organizational barriers.

### 2.2. Setting

The study was conducted in public primary schools in Poland under routine school conditions. The intervention was delivered in classrooms during scheduled school lessons to classes enrolled in the program by school principals. The program constituted part of local government activities in the field of child and adolescent health promotion and was implemented within the framework of the Municipal Nutrition Education Program coordinated by the Social Communication Center of the City of Warsaw [19].

### 2.3. Participants

At the beginning of the 2024/2025 school year, all public primary schools operating within the city of Warsaw (*n* = 224) were invited to participate in the program, providing citywide coverage at the invitation stage. Participation was voluntary and invitation-based; therefore, the final sample consisted of schools that actively enrolled in the program. Ultimately, 81 schools participated. As a result, participating schools may have differed from non-participating schools in terms of organizational readiness, interest in nutrition education, or general health-promotion orientation.

The intervention was delivered under typical classroom conditions in the natural school environment. Workshops were conducted in person on school premises and targeted second-grade primary school students. On average, sessions were delivered in three class groups per school.

A total of 198 workshop cycles were delivered by six trained program implementers. The process evaluation included 198 evaluation forms completed by class teacher-observers present during the sessions, resulting in data collected from 198 teacher-observers. The target population consisted of second-grade students in the Polish education system (approximately 8–9 years old), corresponding to ISCED level 1.

No individual-level demographic data were collected from students, including sex, socioeconomic status, migration background, or household characteristics. Similarly, no school-level socioeconomic or diversity indicators were available in the implementation records. The available contextual information was limited to the type of schools participating in the municipal program, the number of schools and classes, class size, and the fact that all schools operated within the public primary education system of the City of Warsaw.

**Figure 1:**
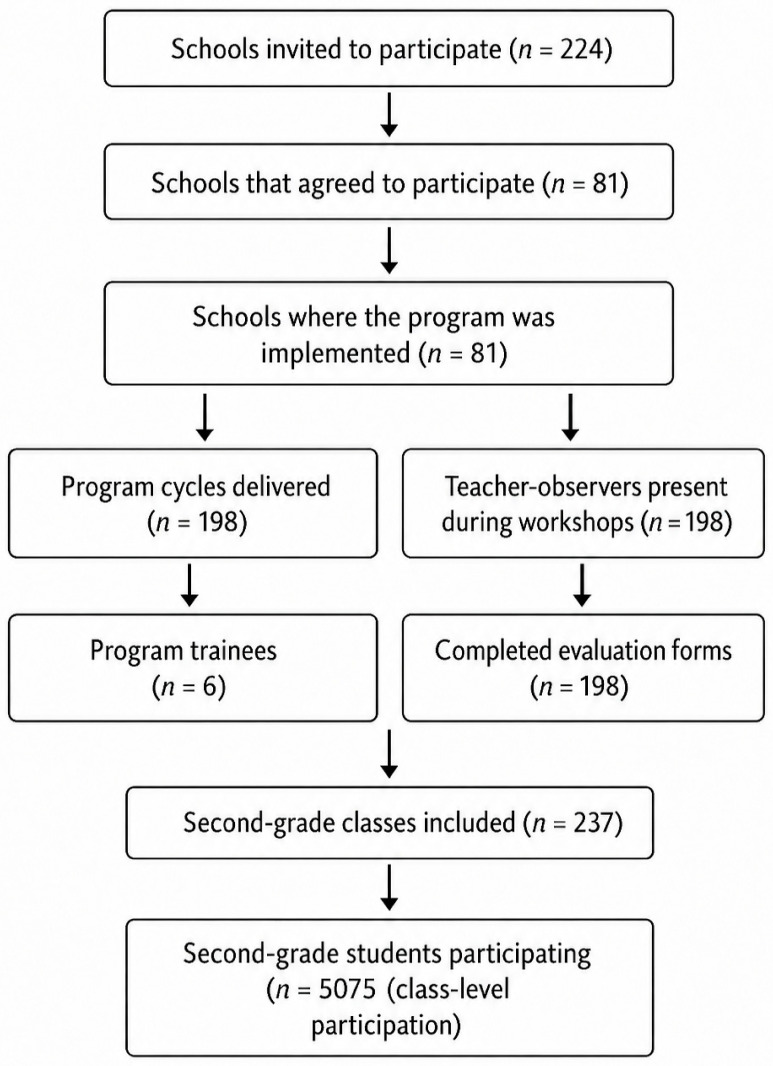
Flow of schools and participants through the school-based nutrition education program.

Recruitment was conducted at the institutional and class levels. Schools enrolled specific class groups, and all students present during the workshops participated in the intervention. The unit of participation and intervention delivery was the school class (class-level participation), while the unit of observation in the process evaluation was the teacher-observer. The flow of schools and participants through the program is presented in Figure 1. All public primary schools in Warsaw were eligible to participate, and no formal exclusion criteria were applied.

The sample size was not determined a priori, as the study followed an exploratory process evaluation design. Instead, it was determined by the number of schools that voluntarily enrolled in the program.

### 2.4. Intervention Description

The structure of the workshops was based on the concept of experiential learning, which emphasizes active student engagement, practical task performance, and reflection on acquired knowledge [8,25]. Additionally, principles of social cognitive theory were incorporated, highlighting the role of behavior modeling, social interactions, and the educational environment in shaping health-related competencies [7].

The intervention also integrated elements of transformative learning theory, according to which changes in attitudes and behaviors are preceded by critical self-reflection on one’s beliefs and prior patterns of action. The inclusion of reflective components was intended to support informed food choices and foster the development of sustainable health competencies among students [26].

“I Know What I Eat” is a municipal nutrition education initiative that has been continuously implemented in primary schools since 2016 [19]. Its aim is to develop children’s nutrition-related knowledge, attitudes, and skills, while also supporting the school food environment through structured health education. The program has been systematically developed and refined through collaboration among the scientific community, practitioners, and local government units.

The intervention was delivered in primary schools to second-grade students in the Polish education system (approximately 8–9 years old). Participation was institutional in nature, with schools enrolling specific class groups. Sessions were conducted in classrooms during routine school activities.

The intervention consisted of a structured cycle of three educational workshops delivered by trained program implementers once a week over three consecutive weeks, integrating both theoretical and practical components. Each workshop lasted one standard class period (45 min), resulting in a total exposure time of 135 min.

Workshop I addressed the fundamentals of healthy eating and the role of breakfast. Workshop II focused on local and seasonal foods and the prevention of food waste. Workshop III had a practical format and aimed to develop students’ skills in preparing simple, healthy meals (Table 1).

All sessions were delivered using student-engaging methods, visual materials, and action-based learning approaches. Standardization of the intervention was ensured through uniform lesson plans, standardized educational materials, and the training of program implementers. No additional strategies to enhance adherence were applied beyond standard classroom engagement and the presence of teacher-observers.

From a behavioral perspective, the intervention was designed to activate mechanisms such as engagement, experiential learning, and social interaction, which may support the development of food-related decision-making processes.

### 2.5. Program Implementers

The intervention was delivered by six trained program implementers with a background in dietetics, who conducted workshops in primary schools. The group consisted of five women and one man.

All sessions were delivered in accordance with standardized instructional protocols and uniform educational materials, ensuring consistency of content and a consistent standard of implementation. The program implementers were responsible for delivering educational content, applying student-engaging methods, and organizing the course of the sessions in line with the program framework.

### 2.6. Training of Program Implementers

Program implementers were prepared to deliver the intervention through an intensive, in-person training conducted over five days (8 h per day) prior to program implementation in schools. The training included both theoretical and methodological components and was delivered by a multidisciplinary team comprising a health education researcher and three practitioner-educators with experience in school-based interventions.

The training covered the theoretical framework of the program, the content and structure of individual workshops, methods for working with children, and organizational aspects of session delivery in school settings. Program implementers were also instructed in the use of student-engaging methods and standardized instructional protocols. The training aimed to ensure consistency of program delivery, a uniform standard of implementation, and fidelity to the intervention design.

### 2.7. Process Evaluation

#### 2.7.1. Scope of the Process Evaluation

The process evaluation used a multidimensional approach to assess the quality of intervention delivery in routine school practice. Its purpose was to provide an in-depth understanding of how the program was implemented, how it was received, and how it may have activated mechanisms relevant to children’s food choice behaviors and food-related decision-making.

For clarity, the term “teacher-observers” is used throughout the manuscript to refer to classroom teachers who were present during the workshops and completed the evaluation forms, whereas the term “program implementers” refers to the trained individuals responsible for delivering the workshops.

One of the primary sources of evaluation was the perspective of teacher-observers present during the sessions. These teacher-observers were experienced educators working daily with children in grades 1–3 of primary school, supporting the validity of their assessments of the program’s suitability to students’ developmental needs. From this perspective, the quality of session delivery, the appropriateness of educational content, the level of student engagement, the organization of the workshops, and the overall acceptability of the intervention were assessed. Qualitative data on program strengths and areas for improvement were also collected.

The second source of evaluation was the perspective of program implementers. This included reflections on session delivery, organizational aspects of implementation within the school environment, adherence to standardized protocols, and experiences related to delivering the intervention in everyday classroom practice.

#### 2.7.2. Process Evaluation Outcomes

The primary outcomes of the process evaluation were implementation quality and implementation fidelity, while secondary outcomes included acceptability, organizational feasibility, and perceived strengths and areas for improvement. The evaluated domains reflect key dimensions of process evaluation in complex school-based educational interventions.

The main process evaluation indicators included implementation quality, assessed based on teacher-observers’ evaluations of the educational value of the workshops, clarity of content, appropriateness to students’ developmental level, quality of program implementer–student interactions, and the usefulness of educational materials. Implementation fidelity was defined as the extent to which the workshops were delivered in accordance with standardized protocols and the program’s methodological assumptions. Acceptability was assessed based on teachers’ overall evaluation of the workshops and their perceived usefulness within the school context. Organizational feasibility referred to the practical feasibility of delivering the intervention under typical school conditions. Perceived strengths and areas for improvement were identified based on qualitative feedback provided by teacher-observers and program implementers.

These domains were also interpreted in relation to their potential role in shaping food choice drivers, including engagement, experiential learning, social interaction, and processes related to food-related decision-making.

#### 2.7.3. Data Collection Tools

The quality of program delivery was assessed using an author-developed workshop evaluation form completed by teacher-observers present during the sessions. The instrument included closed-ended items rated on a 5-point Likert scale, as well as open-ended questions enabling the collection of qualitative feedback.

The questionnaire consisted of a general section and modules corresponding to each of the three workshop components. Within the module-specific sections, teacher-observers assessed the usefulness of workshop topics, the appropriateness of content for students’ age, and the practical relevance of the material. The general section included evaluation of implementers’ subject-matter preparation, the quality of interactions with students, and the usefulness of educational materials.

Qualitative responses were analyzed using thematic content analysis, combining predefined analytical domains with inductively derived themes emerging from the data. The resulting themes were subsequently used to interpret patterns observed in the qualitative findings.

The instrument underwent expert review and pilot testing to assess content validity and usability. Its development was grounded in established process evaluation frameworks and aligned with the objectives of the program; however, it was not a previously validated standardized tool.

Prior to implementation, the instrument was pilot-tested in selected educational settings to assess clarity and usability. Revisions included refinement of item wording and questionnaire structure (see Supplementary Material S1).

Because the evaluation form was designed as a pragmatic process evaluation tool rather than as a psychometric scale measuring a single latent construct, formal psychometric validation was not performed. Internal consistency coefficients were not calculated, as the items assessed distinct process-related domains, including usefulness of workshop topics, age appropriateness, practical relevance, program implementer preparation, quality of interaction, and usefulness of educational materials. Similarly, test–retest reliability was not assessed because the instrument was completed once after the workshop cycle and was intended to capture immediate implementation-related perceptions. Therefore, the instrument should be interpreted as an author-developed process evaluation tool with content and face validity supported by expert review and pilot testing, rather than as a formally validated outcome measure.

The interpretation of process evaluation outcomes was informed by conceptual models of behavior change and food choice, including the interaction of cognitive, social, and environmental determinants described in contemporary food choice frameworks.

### 2.8. Data Analysis

Quantitative data were analyzed using descriptive statistics. Measures of central tendency and response ranges were calculated to summarize evaluations of individual workshop components. Qualitative data derived from open-ended questions were analyzed using thematic content analysis. Responses were coded and categorized using a combination of predefined analytical domains and inductively derived themes emerging from the data. This approach allowed for both structured evaluation and the identification of unanticipated patterns.

The overall analytical approach had a descriptive–interpretative character. Data triangulation was applied by integrating quantitative and qualitative findings to provide a comprehensive understanding of the intervention implementation process.

Inferential statistical methods were also used in the quantitative analysis. Differences between program implementers were assessed using the nonparametric Kruskal–Wallis test, due to the ordinal nature of the data and the lack of normal distribution. Associations between class size and workshop quality ratings were examined using Spearman’s rank correlation coefficient. The strength of associations was interpreted according to commonly accepted thresholds. Statistical significance was set at *p* < 0.05.

Effect sizes were calculated for all inferential analyses. For Kruskal–Wallis tests, eta-squared based on the H statistic was calculated using the formula η^2^H = max [0, (H − k + 1)/(N − k)], where H is the Kruskal–Wallis test statistic, k is the number of groups, and N is the analysis-specific sample size. The 95% confidence intervals for η^2^H were estimated using stratified non-parametric bootstrapping with 5000 resamples. For Spearman correlation analyses, rho coefficients were treated as effect size estimates and reported with approximate 95% confidence intervals calculated using Fisher’s z transformation.

Primary statistical analyses were conducted using SPSS Version 11.0 for Windows (IBM Corp., Armonk, NY, USA). Effect size estimates and bootstrap confidence intervals for η^2^H were calculated using an additional resampling procedure. The primary statistical analyses were conducted in accordance with a predefined analytical plan. Effect size estimates and confidence intervals were added during revision to improve the transparency and interpretability of statistical reporting.

The analytical approach also aimed to identify patterns in the data that could inform the understanding of mechanisms influencing food-related decision-making processes.

## 3. Results

### 3.1. Scale and Characteristics of Program Delivery

The program was implemented in 81 schools, with a total of 198 workshop cycles delivered. All evaluation forms (*n* = 198) were included in the analysis, with no missing data identified. The workshops were conducted in 237 classes, involving a total of 5075 students.

All workshops were delivered in accordance with standardized instructional protocols, indicating high implementation fidelity. As the study focused on process evaluation during intervention delivery, no follow-up or attrition was applicable.

No individual-level demographic data or school-level socioeconomic indicators were collected, as the analysis was conducted at the class and program levels using routine implementation records. Therefore, the study does not allow comparison of implementation quality across student demographic subgroups or school socioeconomic contexts.

### 3.2. Assessment of Program Delivery Quality—Quantitative Analysis

#### 3.2.1. Workshop I

Workshop I received very high ratings for both protocol quality (mean = 4.96, SD = 0.24) and delivery quality (mean = 4.94, SD = 0.31). No statistically significant differences were observed between program implementers for either the protocol evaluation (H(5) = 0.50; *p* = 0.992) or delivery (H(5) = 9.46; *p* = 0.092).

#### 3.2.2. Workshop II

Workshop II was also rated highly in terms of both protocol quality (mean = 4.92, SD = 0.30) and delivery quality (mean = 4.91, SD = 0.32). No statistically significant differences were found between implementers for protocol evaluation (H(5) = 5.03; *p* = 0.413) or delivery (H(5) = 4.57; *p* = 0.470).

#### 3.2.3. Workshop III

Workshop III received high ratings for both protocol quality (mean = 4.94, SD = 0.31) and delivery quality (mean = 4.88, SD = 0.45). No statistically significant differences were observed between implementers for protocol evaluation (H(5) = 6.84; *p* = 0.233) or delivery (H(5) = 5.44; *p* = 0.365).

#### 3.2.4. Overall Comparison

Across all workshops, no statistically significant differences were observed between program implementers across any evaluated dimension (all *p* > 0.05), indicating a high level of consistency in intervention delivery. Effect size estimates were negligible to small, with η^2^H values ranging from 0.000 to 0.023. The lower bounds of the 95% confidence intervals for η^2^H were 0.000 across all evaluated dimensions, and the upper bounds remained low, supporting the interpretation that differences between program implementers explained only a very small proportion of variability in workshop ratings (Table 2).

### 3.3. Comparison of Modules

All workshop modules received consistently high ratings for both instructional protocol quality and delivery. Mean scores ranged from 4.88 to 4.96 on a five-point scale, indicating a very high level of evaluation across all modules.

For instructional protocol quality, mean ratings ranged from 4.92 (SD = 0.30) for Workshop II to 4.96 (SD = 0.24) for Workshop I (and for Workshop III: 4.94, SD = 0.31). For delivery, mean ratings ranged from 4.88 (SD = 0.45) for Workshop III to 4.94 (SD = 0.31) for Workshop I, with Workshop II rated at 4.91 (SD = 0.32). Standard deviation values were low across all modules, indicating limited variability in responses.

Ratings were concentrated at the upper end of the scale, with most responses falling between 4 and 5 points. This distribution indicates limited variability in responses and suggests that the scale may have had limited ability to discriminate between different levels of perceived implementation quality. No statistically significant differences were observed between program implementers across any of the analyzed dimensions (all *p* > 0.05), indicating a high level of consistency in workshop delivery.

Overall, the findings indicate that all modules were delivered at a comparably high level. The consistently high ratings, particularly for modules incorporating active participation and practical activities, point to the importance of process-related factors such as student engagement and experiential learning in supporting food-related decision-making processes.

### 3.4. Effect of Class Size on Quality Ratings

Spearman’s rank correlation analysis showed no statistically significant associations between class size and workshop ratings across all modules and evaluated dimensions. Correlation coefficients were very weak, with ρ values ranging from −0.071 to 0.118, and all 95% confidence intervals included zero. These findings indicate no meaningful relationship between class size and evaluations of both protocol quality and delivery quality. The mean class size was 22 students (SD = 3.36; range: 7–30). Detailed correlation results are presented in Table 3.

### 3.5. Qualitative Analysis of Teacher-Observer Comments on Program Delivery

Qualitative data provided by teacher-observers were analyzed to assess perceptions of workshop implementation. The responses included both overall evaluations and detailed comments regarding session organization, delivery, and student engagement.

Across all modules, positive evaluations predominated, highlighting the appropriateness of the content, high levels of student engagement, and the overall quality of session delivery. Only a minority of comments indicated areas for improvement, primarily related to organizational aspects such as session duration, the inclusion of movement-based activities, and the need for further enhancement of student-engaging elements.

Overall, the qualitative findings indicate high acceptability of the program and a consistently positive reception of the workshops among teacher-observers.

#### 3.5.1. Workshop I—Thematic Analysis

The qualitative analysis of 198 comments indicates a predominantly positive distribution of responses. The majority of comments (80.8%, *n* = 160) were positive and emphasized the high quality of session delivery, the professionalism of program implementers, and strong student engagement. The remaining responses (19.2%, *n* = 38) were neutral and primarily included suggestions for improvement, such as increasing the number of practical activities, introducing short movement breaks, and reducing lecture-based elements. No clearly negative comments were identified.

The thematic analysis identified four main categories: (1) high quality and professionalism of session delivery; (2) strong student engagement and the use of interactive teaching methods; (3) appropriateness of content to participants’ developmental level and a positive classroom atmosphere; and (4) constructive suggestions for improvement. Overall, the qualitative patterns suggest very high acceptability of the intervention, with feedback being predominantly developmental rather than critical in nature.

Selected representative quotes from teacher-observers illustrating the main thematic areas are presented in Table 4. The findings highlight the high quality of delivery of the theoretical module and its strong acceptability within the school setting, particularly in relation to student engagement and the use of interactive learning approaches.

#### 3.5.2. Workshop II—Thematic Analysis

The qualitative analysis of 198 comments related to Workshop II indicates a predominantly positive distribution of responses. The majority of comments (83.8%, *n* = 166) were positive and emphasized the high quality of session delivery, strong student engagement, and the practical, workshop-based format of the sessions. The remaining responses (16.2%, *n* = 32) were neutral and primarily included suggestions for improvement, such as increasing the number of activities, optimizing the organization of group work, and expanding the thematic scope. No clearly negative comments were identified.

The thematic analysis identified three main categories: (1) high quality and professionalism of session delivery; (2) strong student engagement, particularly through practical activities; and (3) constructive suggestions for improvement. The practical nature of the workshop emerged as a key factor supporting active student participation and direct engagement with food-related content.

Selected representative quotes from teacher-observers illustrating the main thematic areas are presented in Table 5. Overall, the qualitative patterns indicate very high acceptability of Workshop II, with feedback being predominantly improvement-oriented. The strong emphasis on hands-on activities highlights experiential learning as an important process element supporting engagement and food-related decision-making.

#### 3.5.3. Workshop III—Thematic Analysis

The qualitative analysis of 198 comments related to Workshop III indicates a clearly positive reception of the sessions. The majority of comments (79.8%, *n* = 158) were positive and emphasized the high quality of delivery, the attractiveness of the sessions, and the effective integration of educational content with physical activity. The remaining responses (20.2%, *n* = 40) were neutral and primarily included suggestions for improvement. No clearly negative comments were identified.

The thematic analysis identified three main categories: (1) high quality and attractiveness of the workshop; (2) strong student engagement; and (3) constructive suggestions for improvement. The most frequently reported suggestion concerned extending session duration, indicating that a standard class period (45 min) was insufficient to fully implement the planned activities.

Selected representative quotes from teacher-observers illustrating the main thematic areas are presented in Table 6. The culinary module demonstrated the highest levels of student engagement and a strong practical dimension of the intervention. Overall, the findings indicate high acceptability of Workshop III, with limitations primarily related to time constraints rather than the quality of content or delivery. The strong emphasis on hands-on activities highlights the importance of experiential learning, active participation, and practical engagement as process elements related to children’s food-related decision-making.

The integration of quantitative and qualitative findings indicated consistent patterns, particularly highlighting the role of student engagement and experiential learning across data sources.

### 3.6. Perspective of Program Implementers

An additional component of the process evaluation involved analysis of the experiences of program implementers. The empirical material included self-reflections and insights from a focus group conducted after completion of the workshop cycle.

Program implementers consistently emphasized that the highest levels of student engagement were observed during activities requiring active participation, particularly practical tasks and interactive components. Activities such as preparing meals, handling food products, and incorporating playful elements supported sustained attention and enhanced motivation. As one program implementer noted: “Children were most engaged in activities they could perform independently—cutting, mixing, arranging ingredients. Their pride in the outcomes of their work was clearly visible (Program implementer 1).”

The observations also highlighted the importance of social mechanisms in shaping behavior. Program implementers reported that willingness to try new foods often spread within the group, suggesting a social modeling effect: “When one child decided to try a new product, the rest of the class immediately became more willing. It worked like a domino effect (Program implementer 2).” This pattern reflects the potential role of peer influence as a driver of food-related behaviors in group settings.

The school environment was identified as a facilitating factor, providing a familiar context that supported engagement and a sense of safety, particularly in the presence of a teacher-observer. At the same time, program implementers emphasized the importance of flexibility in adapting activities to students’ developmental levels and group dynamics.

Organizational challenges were also reported. Time constraints were the most frequently identified limitation, particularly in the culinary module, where preparation, tasting, and clean-up had to be completed within a single lesson: “The most difficult part was fitting everything into a single lesson—preparation, eating, and cleaning. This module definitely needs more time (Program implementer 3).”

Additional challenges included adapting sessions to diverse student needs and ensuring adequate logistical preparation, including workspace organization and availability of materials. Program implementers also highlighted the importance of clearly structuring sessions and communicating activity stages to maintain student focus: “When we clearly explained at the beginning what we would be doing and what dish we would prepare, the children worked more calmly and were more focused (Program implementer 4).”

The reflections also indicated the development of program implementers’ pedagogical competencies, including increased self-confidence, improved management of group dynamics, and greater flexibility in responding to unexpected situations. The use of standardized protocols, combined with opportunities for reflection and experience sharing, supported continuous improvement in teaching practice. Suggestions for further program development included expanding content related to healthier alternatives to highly processed foods and strengthening practical, student-engaging components.

No adverse events or unintended negative effects were reported during the implementation of the intervention.

## 4. Discussion

### 4.1. Interpretation of Main Findings

The present study provides empirically grounded process-evaluation evidence on mechanisms potentially related to children’s food choice behaviors. By integrating perspectives from teacher-observers and program implementers with quantitative implementation indicators, the study shows how the intervention was delivered and which process elements were linked to food-related engagement and decision-making.

The findings indicate that the program was delivered with high quality, consistently across program implementers, and was acceptable within the school environment, while also being organizationally feasible at scale. However, the very high ratings observed across all workshop components should be interpreted with caution. Mean scores close to the upper limit of the scale, together with low standard deviations, may indicate a ceiling effect and limited discriminative sensitivity of the evaluation instrument. In other words, the instrument may have been better suited to identifying major implementation problems than to differentiating between more subtle variations in delivery quality. Additionally, because the ratings were provided by teacher-observers present during the sessions, social desirability bias and observer bias may have contributed to uniformly positive assessments. These factors should be considered when interpreting the consistency of the findings and the lack of statistically significant differences between program implementers. It should also be noted that the program has been implemented and systematically developed over a period of approximately 10 years. This long-term implementation may have contributed to the refinement of both the content and delivery procedures, potentially resulting in a mature, well-optimized intervention model and consistently high quality ratings observed in the present study.

Despite these limitations, the results suggest that the intervention was implemented under conditions conducive to student engagement, which is recognized as one of the key determinants of the effectiveness of educational interventions aimed at changing dietary behaviors [5,17]. These findings indicate that experiential learning and social interaction were important process elements that may support engagement and potential behavior change mechanisms.

### 4.2. Comparison with Similar Studies and Unique Contribution

The findings of the present study are consistent with previous school-based nutrition and food education interventions showing that implementation quality, acceptability, and contextual feasibility are central to understanding how programs operate in school settings [27,28,29,30,31]. For example, the FEAST program, a 10-week curriculum-aligned intervention integrating healthy eating, food waste, sustainability, and cooking skills, was well received by students and teachers, although implementation fidelity was limited by COVID-19-related school closures and the study did not demonstrate significant improvements in fruit and vegetable intake or other primary or secondary outcomes [27]. This supports the importance of distinguishing between process-level acceptability and demonstrated behavioral effectiveness. In the present study, similarly high acceptability and positive perceptions of practical components were observed, but the process-oriented design does not allow conclusions about direct dietary behavior change.

The present findings also correspond with the process evaluation of Project DAIRE, which identified social learning, experiential learning, interactive engaging content, and real-life connections as key mechanisms of impact in a school-based food environment and food education intervention [28]. These mechanisms are closely aligned with the qualitative findings of the present study, in which teacher-observers and program implementers emphasized active participation, practical food preparation, peer interaction, and student engagement as central elements of the intervention. Similarly to DAIRE, time constraints emerged as an important contextual issue. In the present study, this barrier was particularly evident in relation to the practical culinary component [28]. This convergence suggests that experiential and socially embedded learning processes may be important mechanisms across different school-based food education programs.

Other school-based nutrition and health-promotion interventions, including both large multi-site initiatives and smaller whole-school programs, also highlight the importance of implementation conditions. The HEALTHY nutrition intervention demonstrated that process evaluation is useful for documenting whether intervention components are delivered as intended and for identifying barriers such as costs, food availability, and student acceptance [29]. Similarly, the APPLES program showed that school-based health promotion requires attention to implementation quality, teacher training, school-level support, and institutional ownership of action plans [30]. In contrast, the present intervention was not primarily focused on changing the total school food environment but on structured educational delivery by trained program implementers. Nevertheless, these studies underline that school-based nutrition interventions require attention not only to intervention content but also to fidelity, feasibility, stakeholder engagement, and contextual constraints.

The study also differs from dissemination-oriented programs such as Shaping Up My Choices, which used the RE-AIM framework to assess reach, efficacy, adoption, implementation, and maintenance across a large population of third-grade students [31]. While that program demonstrated broad dissemination potential, the present study contributes a different type of evidence by examining a municipal nutrition education program implemented under routine school conditions across 81 schools, 198 workshop cycles, 237 classes, and 5075 students. This scale strengthens the ecological relevance of the findings and provides insight into how standardized educational content can be delivered consistently across multiple schools and program implementers.

A further unique contribution of this study is the integration of process evaluation with a food choice and consumer behavior perspective. Previous studies have often focused on dietary intake, nutrition knowledge, food environment change, implementation fidelity, or public health impact [27,28,29,30,31]. In contrast, the present study interprets implementation findings in relation to potential drivers of children’s food-related decision-making. Children are therefore conceptualized not only as recipients of nutrition education but also as emerging consumers whose early experiences with food, peer interaction, practical preparation, and perceived competence may shape future food preferences and decision-making patterns. This perspective broadens the interpretation of school-based nutrition education beyond knowledge transfer and highlights its potential role in activating cognitive, social, behavioral, and environmental drivers of food choice.

### 4.3. Mechanisms Underlying Food Choice Behaviors

The findings can be interpreted as process-level evidence of several mechanisms potentially relevant to children’s food choices. In particular, the results highlight the role of (1) social drivers, such as peer influence and modeling; (2) behavioral drivers, including the development of practical skills and hands-on experience; (3) cognitive drivers, related to knowledge acquisition and awareness; and (4) environmental drivers, associated with the school setting as a structured food environment. These interacting dimensions reflect the multidimensional nature of food choice behaviors described in the literature. These findings align with broader conceptualizations of food choice as a form of consumer behavior shaped by interacting cognitive, social, and environmental influences. Importantly, these findings extend beyond individual behavior and may have implications for food systems, as early-life food choice drivers contribute to shaping long-term consumer demand patterns and dietary trends at the population level.

### 4.4. Implementation Quality and Intervention Delivery

The high ratings across all workshop modules indicate very good implementation quality, regardless of the thematic component. Standardized instructional protocols, training of program implementers, and substantive supervision likely enabled the maintenance of fidelity to the program’s assumptions despite delivery across diverse school settings. In addition, the long-term implementation of the program (approximately 10 years) may have contributed to the development of a mature and well-standardized intervention model, supporting consistent delivery and high implementation fidelity across settings. From an implementation science perspective, this is particularly important, as maintaining implementation quality during scale-up represents one of the key challenges of population-level programs [17,25].

In the context of educational interventions, high implementation quality appears to be not only a prerequisite for consistent delivery but also a condition that enables processes relevant to dietary behavior change.

### 4.5. Role of Educational and Behavioral Theories

The interpretation of the findings is consistent with the assumptions of the educational theories underpinning the intervention design. The workshop structure, based on experiential learning, promoted active student participation, reinforcement of knowledge through action, and the integration of educational content with direct experience [8,25]. Practical components and interactive tasks were particularly important, as they enabled learning through action, observation, and reflection. This type of approach supports knowledge acquisition and may also strengthen perceived competence and a sense of agency in making food choices [5,7], which is recognized as an important mechanism of health behavior change. In this context, experiential learning can be understood as a behavioral mechanism that links knowledge acquisition with food-related decision-making.

At the same time, the incorporation of elements of social cognitive theory supported the modeling of healthy dietary behaviors and learning through observation and social interactions [7]. Such dynamics illustrate how peer influence may function as a social driver of food choice, particularly in group-based educational settings. The inclusion of narrative-based instructional elements may have enhanced students’ identification with characters and facilitated the application of conveyed content to their own experiences. Evidence suggests that storytelling-based strategies can increase audience engagement and support the acquisition of health-related knowledge as well as the development of health competencies [32,33,34].

The inclusion of reflective elements is consistent with the principles of transformative learning, according to which sustained changes in attitudes and behaviors can be supported through reflection on one’s own experiences [26]. This approach facilitates the transition from declarative knowledge to more enduring changes in attitudes and behaviors by promoting active processing of content and its application to personal experience [35,36,37].

### 4.6. Importance of Practical Components and Student Engagement

One of the most important findings of the study is the significance of student-engaging methods and practical components. Both quantitative and qualitative data indicated that activities requiring active participation were particularly engaging for students. The culinary module, in which children independently prepared meals, played a particularly important role. Such activities function as a bridge between knowledge and action by enhancing motivation, attention, and perceived competence, thereby supporting the internalization of content and its potential application in everyday life [36,38].

### 4.7. Organizational and Contextual Factors

The organizational feasibility of the program is also noteworthy. The lack of association between class size and workshop quality ratings suggests that group size itself was not a key determinant of implementation quality. This finding indicates that the quality of instructional interactions may be more important than organizational parameters. The literature emphasizes that the quality of teacher–student relationships is a significant predictor of student engagement and social functioning [39]. At the same time, the identified barriers, such as time constraints and logistical challenges, point to the need for further refinement of implementation conditions, particularly with regard to practical components [24].

A particularly important organizational implication concerns the duration of the culinary workshop. Both teacher-observers and program implementers indicated that the 45 min duration of the culinary workshop was insufficient to complete all stages of the practical module, including preparation, food handling, tasting, reflection, and clean-up. Future implementation should therefore consider extending the culinary workshop or dividing it into two separate sessions. This adjustment could reduce time pressure, improve safety and organization, and allow students to engage more fully in the experiential learning process.

In addition to time constraints, logistical and material preparation should be considered a key condition for high-quality implementation of practical nutrition education. The availability of appropriate equipment, sufficient workspace, and clearly organized materials may facilitate smoother session delivery and reduce the burden on program implementers. In culinary workshops involving young children, these aspects are also directly linked to safety, hygiene, and the quality of student participation. Strengthening logistical procedures before each session may therefore improve both implementation fidelity and the educational value of the practical component.

### 4.8. Strengths of the Study

An important strength of the study was the use of a multi-perspective process evaluation. The convergence of assessments from teachers and program implementers strengthens the credibility of the findings and reduces the risk of one-sided interpretation. At the same time, the complementarity of these perspectives allows for a better understanding of how organizational conditions and the quality of instructional interactions jointly shape an environment conducive to effective intervention delivery. This approach is consistent with recommendations from implementation research, which emphasize the importance of data triangulation in evaluating health interventions [17,24].

The findings of the study also have system-level implications. The program represents an example of an intervention developed through collaboration between the scientific community, practitioners, and local government administration, which enables alignment with institutional realities and enhances sustainability. Moreover, the long-term implementation of the program (approximately 10 years) represents an additional strength, as it reflects sustained integration within the educational system and supports the ecological validity of the findings in routine educational practice. Such an approach may facilitate the effective implementation of interventions at the population level and support the promotion of healthy behaviors among children [40].

### 4.9. Limitations

When interpreting the findings, several limitations of the study should be considered. The evaluation had a process-oriented design and did not include direct measurement of changes in dietary behaviors or long-term health outcomes. Therefore, the results should be interpreted as reflecting implementation conditions and processes that may precede behavior change, rather than as direct evidence of intervention effectiveness. The findings suggest that experiential learning and active participation may support mechanisms such as engagement, social modeling, and perceived self-efficacy, which are considered important determinants of dietary behavior change. Additionally, the assessments were based on the perspectives of teachers and program implementers, which is associated with a risk of subjectivity, and the study had a cross-sectional design. The reliance on teacher-reported outcomes may introduce observer bias and social desirability bias, potentially leading to overestimation of intervention quality.

Another important limitation is the absence of validated outcome measures and direct behavioral assessment. The study did not include standardized measures of children’s nutrition knowledge, food literacy, self-efficacy, food preferences, dietary intake, or observed food choice behaviors. Instead, the evaluation relied on process indicators and teacher-observer and program implementer perspectives, which provide valuable information on implementation quality, acceptability, and potential mechanisms, but do not allow conclusions regarding actual behavioral change. Therefore, the identified drivers should be interpreted as potential mechanisms inferred from implementation processes rather than as directly measured determinants of children’s food choices. Future studies should incorporate validated child-level outcome measures, direct assessment of food choices or dietary intake, and longitudinal follow-up to examine whether the observed process mechanisms translate into sustained behavioral outcomes. In addition, the author-developed process evaluation form was not formally validated, and its psychometric properties, including internal consistency and test–retest reliability, were not established.

The study was limited by its non-randomized, single-group design, which restricts causal inference and limits the ability to attribute observed outcomes directly to the intervention. Another important limitation concerns the voluntary, invitation-based recruitment of schools. Although all public primary schools in Warsaw were invited to participate, only 81 schools enrolled in the program. Schools that voluntarily joined the intervention may have been more proactive, better organized, or more interested in nutrition education and health promotion than non-participating schools. This self-selection may have contributed to the high acceptability ratings and positive implementation outcomes observed in the study. Consequently, the findings should not be interpreted as representative of all primary schools in Warsaw or Poland, but rather as reflecting implementation conditions among schools willing to participate in a municipal nutrition education program. In addition, the lack of student-level demographic data and school-level socioeconomic indicators limits the ability to assess whether the implementation process differed according to student characteristics, school diversity, or socioeconomic context. Future evaluations should include contextual indicators such as school district, neighborhood deprivation, school size, proportion of students receiving social support or free meals, and student-level demographic variables, where ethically and legally appropriate.

### 4.10. Implications for Practice and Future Research

The findings of this study have several implications for the design and implementation of nutrition education programs in school settings. The results indicate that standardized instructional protocols, structured training of program implementers, and institutional embedding of the intervention are important conditions for consistent and scalable implementation in routine school practice.

In particular, the findings suggest that the introductory module could benefit from a greater variety of hands-on and movement-based activities. Although Workshop I was evaluated very positively, qualitative comments indicated that some students may benefit from more opportunities for independent task performance rather than primarily listening and observing. Therefore, future editions of the program should consider strengthening the practical and interactive components of the first workshop, for example by incorporating short movement-based tasks, food classification exercises, or small-group activities. Such modifications may further enhance student engagement and ensure stronger alignment between the theoretical introduction and the experiential learning principles underpinning the program.

The findings also indicate that student-engaging methods and practical components, particularly those based on active participation and experiential learning, are closely linked to engagement and to processes described in the literature as relevant to health behavior change. The integration of theoretical content with practical experience may be an important element in facilitating the transition from knowledge acquisition to potential behavioral application.

From a broader perspective, the program represents an intervention implemented through collaboration between academic, practice, and local government stakeholders, supporting alignment with organizational contexts and facilitating scalability.

From a food systems and consumer behavior perspective, early-life interventions implemented in educational settings are relevant to the development of food choice drivers that may persist over time. By targeting experiential, social, and cognitive dimensions, such programs may influence how children perceive, evaluate, and select foods within their everyday environments.

However, these implications should be interpreted with caution. Due to the process-oriented design of the study and the lack of direct behavioral outcome measures, the findings relate to implementation conditions and potential mechanisms rather than to demonstrated effectiveness. Future research should include the assessment of dietary behaviors and long-term outcomes to further evaluate the impact of such interventions. The findings may be generalizable to similar public school settings operating under structured educational systems and supported by institutional programs; however, caution is warranted when extrapolating the results to different educational or cultural contexts.

## 5. Conclusions

This study provides real-world process evaluation evidence on mechanisms potentially related to children’s food choice behaviors identified through the implementation of a school-based nutrition education program. The findings demonstrate that structured and standardized interventions incorporating experiential learning and practical activities can be delivered consistently across schools and program implementers within routine school practice.

The results highlight the potential role of active participation, practical tasks, and social interaction as key mechanisms supporting food-related decision-making processes, including engagement, social modeling, and perceived self-efficacy. These findings underscore the importance of integrating experiential and behaviorally oriented components into nutrition education programs targeting children.

Due to the process-oriented design, the findings should be interpreted as reflecting potential drivers and mechanisms rather than direct evidence of changes in food choices or dietary intake. However, the scale and long-term municipal implementation of the program support the relevance of these findings for practice and policy. Future research should incorporate direct measures of food-related behaviors and controlled study designs to further examine the role of these mechanisms in shaping long-term dietary outcomes.

## Figures and Tables

**Table 1:** Educational modules and their theoretical foundations within the school-based nutrition education program.

Workshop	Description	Theoretical Foundations
Workshop I: Fundamentals of Healthy Eating and the Role of Breakfast	The workshop aimed to familiarize students with the principles of healthy eating, the structure of the Healthy Eating Pyramid, and the concept of the Healthy Eating Plate, as well as to raise awareness of the importance of regular breakfast consumption. The session included the presentation of educational content, knowledge-reinforcement exercises, and interactive tasks engaging students in the learning process.	Experiential Learning—active acquisition of knowledge through interactive tasks and reinforcement exercises Social Cognitive Theory—modeling of healthy dietary patterns and building health competencies Transformative Learning Theory—reflection on one’s own dietary habits and the importance of regular meals
Workshop II: Local and Seasonal Foods and the Prevention of Food Waste	The workshop aimed to develop students’ awareness of food choice, the importance of local and seasonal foods, and the issue of food waste. The session included classification exercises, analysis of food product examples, moderated discussion, and activating tasks supporting learning through action.	Experiential Learning—learning through action and analysis of food product examples Social Cognitive Theory—shaping attitudes through discussion and the influence of social norms Transformative Learning Theory—developing awareness of the consequences of food choices and supporting food-related decision-making
Workshop III: Practical Preparation of Healthy Meals	The workshop was designed as a practical session aimed at developing students’ skills in independently preparing simple, healthy meals suitable for consumption in the school setting. The session included teamwork in preparing dishes, instruction on the safe use of kitchen equipment, tasting of prepared meals, and a structured summary of the session.	Experiential Learning—learning through direct experience and hands-on task performance Social Cognitive Theory—learning through observation and team work Transformative Learning Theory—reflection on one’s sense of agency and the development of perceived competence in healthy eating and food-related decision-making

**Table 2:** Effect sizes and 95% confidence intervals for differences between program implementers.

Outcome	*n*	H	*p*	η^2^H	95% CI for η^2^H
Workshop I. Protocol quality	198	0.496	0.992	0.000	[0.000, 0.053]
Workshop I. Delivery quality	198	9.459	0.092	0.023	[0.000, 0.138]
Workshop II. Protocol quality	198	5.026	0.413	0.000	[0.000, 0.091]
Workshop II. Delivery quality	198	4.571	0.470	0.000	[0.000, 0.099]
Workshop III. Protocol quality	198	6.835	0.233	0.010	[0.000, 0.084]
Workshop III. Delivery quality	198	5.437	0.365	0.002	[0.000, 0.096]

Note. H = Kruskal–Wallis test statistic; η^2^H = eta-squared based on the H statistic; CI = confidence interval; *n* = number of valid observations included in the analysis. The 95% confidence intervals for η^2^H were estimated using stratified non-parametric bootstrapping with 5000 resamples. Values of η^2^H close to zero indicate negligible differences between program implementers.

**Table 3:** Spearman correlations between class size and workshop ratings.

Outcome	*n*	ρ	*p*	95% CI for ρ
Workshop I. Protocol quality	198	−0.071	0.321	[−0.208, 0.069]
Workshop I. Delivery quality	198	−0.031	0.669	[−0.169, 0.109]
Workshop II. Protocol quality	198	0.074	0.298	[−0.066, 0.212]
Workshop II. Delivery quality	198	0.008	0.913	[−0.132, 0.147]
Workshop III. Protocol quality	198	0.118	0.099	[−0.022, 0.253]
Workshop III. Delivery quality	198	0.040	0.582	[−0.101, 0.179]

Note. ρ = Spearman’s rank correlation coefficient; CI = confidence interval; *n* = number of valid observations included in the analysis. Confidence intervals for ρ were calculated using Fisher’s z transformation. Values close to zero indicate negligible associations between class size and workshop ratings.

**Table 4:** Selected teacher-observer quotes on the implementation of Workshop I.

Thematic Category	Representative Teacher-Observer Quotes
High quality and professionalism of session delivery	“The program implementers were very well prepared in terms of subject matter, established excellent rapport with the class, and demonstrated a high level of professionalism” (Teacher-observer 4).
High student engagement and use of interactive teaching methods	“The sessions were very well organized; the variety of teaching methods helped sustain students’ attention” (Teacher-observer 5).
Appropriateness of content to participants’ developmental level and positive classroom atmosphere	“The content was delivered in a way that was understandable to children and appropriate for their age” (Teacher-observer 8).
Constructive suggestions for improvement	“More tasks involving independent work by the children could be included, rather than relying solely on listening and observing” (Teacher-observer 14).

**Table 5:** Selected teacher-observer quotes on the implementation of Workshop II.

Thematic Category	Representative Teacher-Observer Quotes
High quality and professionalism of session delivery	“The program implementers were able to engage the children in the topic and created a pleasant atmosphere conducive to learning” (Teacher-observer 17).
High student engagement, particularly through practical activities	“Theoretical content was interspersed with practical tasks, which helped maintain students’ attention” (Teacher-observer 20).
Constructive suggestions for improvement	“Children should perform all tasks independently, with support from the program implementers” (Teacher-observer 22).

**Table 6:** Selected teacher-observer quotes on the implementation of Workshop III.

Thematic Category	Representative Teacher-Observer Quotes
High quality and professionalism of session delivery	“The sessions were conducted professionally, with attention to safety and hygiene standards” (Teacher-observer 24).
High student engagement	“Children willingly engaged in cutting ingredients and actively participated in food preparation” (Teacher-observer 25).
Constructive suggestions for improvement	“The duration of the culinary sessions should be extended, as the current time is insufficient for both food preparation and cleaning” (Teacher-observer 30).

## Data Availability

The data presented in this study is available on request from the corresponding author. The data is not publicly available due to privacy and ethical restrictions.

## Supplementary Materials

The following supporting information can be downloaded at: https://www.mdpi.com/article/10.3390/foods15111832/s1, Supplementary Material S1: Workshop evaluation questionnaire.

## Abbreviations

The following abbreviations are used in this manuscript:

MRC	Medical Research Council
TREND	Transparent Reporting of Evaluations with Nonrandomized Designs
TIDieR	Template for Intervention Description and Replication
ISCED	International Standard Classification of Education
