# Supplementary material for: Exploring Drivers of Children’s Food Choices: A Multi-Source Process Evaluation of a School-Based Nutrition Education Program
Source: Foods. 2026 May 22;15(11):1832. doi: 10.3390/foods15111832 (PMC13256692; doi:10.3390/foods15111832)
Supplement: Supplementary file 1 [file foods-15-01832-s001.zip › foods-4323842-supplementary.pdf]

Supplementary Material S1: Workshop evaluation questionnaire

Workshop Evaluation Form
School name and number: ....
Class: ....
Number of students in the class: ....
Teacher's name and surname: ....

Workshop 1: "Healthy Breakfast at Home and School"

1. How do you assess the topic of the workshop "Healthy Breakfast at Home and School" in terms of its usefulness for students?

1	2	3	4	5
Very poor		Difficult to say		Very good

2. In your opinion, was the content presented during the workshop appropriate for the age of the students?

1	2	3	4	5
Definitely NO		Difficult to say		Definitely YES

3. In your opinion, what could be improved in this workshop?

....

Workshop 2: "I Respect Food"

4. How do you assess the topic of the workshop "I Respect Food" in terms of its usefulness for students?

1	2	3	4	5
Very poor		Difficult to say		Very good

5. In your opinion, did the workshop provide practical guidance on respecting food and preventing food waste that students can apply in everyday life?

1	2	3	4	5
Definitely NO		Difficult to say		Definitely YES

6. In your opinion, what could be improved in this workshop?

....

Workshop 3: "I Know, I Can, I Understand" – Culinary Workshop

7. How do you assess the topic of the workshop "I Know, I Can, I Understand" – culinary session, in terms of its usefulness for students?

1	2	3	4	5
Very poor		Difficult to say		Very good

8. In your opinion, did students have the opportunity to acquire practical skills during the culinary workshop?

1	2	3	4	5
Definitely NO		Difficult to say		Definitely YES
